# Unmixing hysteresis loops of the late Miocene–early Pleistocene loess-red clay sequence

**DOI:** 10.1038/srep29515

**Published:** 2016-07-08

**Authors:** Rui Zhang, Cristian Necula, David Heslop, Junsheng Nie

**Affiliations:** 1 MOE Key Laboratory of Western China’s Environmental Systems, College of Earth and Environmental Sciences, Lanzhou University, Lanzhou 73000, China; 2 Faculty of Physics, Paleomagnetic Laboratory, University of Bucharest, N. Balcescu, 1, 010041 Bucharest, Romania; 3 Research School of Earth Sciences, Australian National University, Acton 2601, ACT, Australia

## Abstract

Magnetic paleoclimatic records often represent mixed environmental signals. Unmixing these signals may improve our understanding of the paleoenvironmental information contained within these records, but such a task is challenging. Here we report an example of numerical unmixing of magnetic hysteresis data obtained from Chinese loess and red clay sequences. We find that the mixed magnetic assemblages of the loess and red clay sediments both contain a component characterized by a narrow hysteresis loop, the abundance of which is positively correlated with magnetic susceptibility. This component has grain sizes close to the superparamagnetic/stable single domain boundary and is attributed to pedogenic activity. Furthermore, a wasp-waisted component is found in both the loess and red clay, however, the wasp-waisted form is more constricted in the red clay. We attribute this component to a mixture of detrital ferrimagnetic grains with pedogenic hematite. The abundance of this component decreases from the base to the top of the red clay, a pattern we attribute to decreased hematite production over the Chinese Loess Plateau (CLP) due to long-term climate cooling. This work demonstrates the potential of hysteresis loop unmixing to recover quantitative paleoclimatic information carried by both low and high coercivity magnetic minerals.

Chinese loess and the red clay sequences that underlie them are invaluable sedimentary archives for reconstructing paleoclimatic evolution since the late Miocene^1^,^2^,^3^,^4^,^5^,^6^,^7^,^8^. To identify and characterize different paleoclimate processes requires quantitative methods to decompose the convolved environmental signals archived in the loess and red clay sequences. Rock magnetic properties of Chinese loess and red clay sediments are generally divided into detrital and pedogenic components^9^,^10^,^11^,^12^. Based on remanence unmixing, some studies suggested that in turn these components can be subdivided into a number of subcategories^13^,^14^, for example, pedogenic maghemite (fine particles, with a peak coercivity of ~30 mT), detrital maghemite (peak coercivity of ~110 mT), slightly altered detrital magnetite (peak coercivity of ~80 mT), pedogenic hematite (median coercivity of ~130 mT) and detrital hematite (peak coercivity of ~600 mT). However, these studies used parametric unmixing techniques that model individual magnetic components using pre-defined mathematical functions (e.g., cumulative log-Gaussian (CLG) or skewed generalized Gaussian (SGG) functions). In addition, such parametric approaches are currently only applicable to single coercivity spectra and therefore do not readily provide a consistent unmixing framework for collections of samples.

In recent years, a non-parametric technique has been developed that simultaneously decomposes a collection of isothermal remanent magnetization (IRM) curves into a small number of fixed components (end-members) with varying abundances^15^. The advantage of such an approach is that enables determination of not only the form of coercivity-based end-members, but also their contributions to individual samples based only on IRM acquisition data. This non-parametric unmixing technique has been successfully applied to the late Miocene-early Pleistocene red clay and loess deposits on the CLP^16^. This work revealed that the red clay and Quaternary loess contain a similar pedogenic component, but their detrital components exhibit different degrees of oxidation, interpreted as indicating long-term climate cooling since the late Miocene. The IRM-based technique was not, however, able to extract paleoclimatic information carried by the high coercivity mineral components of the Chinese loess and red clay, which are critical to test the inferences drawn by studies based on ferrimagnetic materials alone^17^.

Magnetic hysteresis data contain information concerning both induced and remanent magnetizations, which can provide important insights into magnetic mineralogy, concentration and domain state (i.e., grain size). In particular, the wasp-waisted shape of some hysteresis loops reflects a bimodal or multimodal population of magnetic grains that have widely differing coercivities^18^. Thus, unmixing hysteresis loops can potentially provide access to paleoclimatic information carried by high coercivity minerals. In this paper, we report hysteresis unmixing results for a suite of loess and red clay samples from the Chaona section^19^ (Fig. 1) and provide new insights into the link between magnetic mineralogy and paleoclimatic evolution during the late Miocene–early Pleistocene.

## Results

Principal component analysis of hysteresis data from the red clay and loess-paleosol samples, reveals that the leading principal component explains >95% of the data variance^20^. This suggests that a two end-member (EM) mixing model will provide a suitable representation of the hysteresis data. In addition, model selection metrics (I_idx_ quantifying the overall monotonicity of the hysteresis branches and C_idx_ quantifying the extent of branch crossovers)^20^ are both close to 1 (Fig. 2), indicating that a two-EM model is physically realistic.

The two EMs obtained by the unmixing model have significantly different forms (Fig. 3). The hysteresis loop of end-member 1 (EM1) (Fig. 3a,c) is wasp-waisted and closes at ~1 T. End-member 2 (EM2) (Fig. 3b,d) contains a “slim” hysteresis loop that closes at ~500 mT. EM2 has effectively the same hysteresis shape and hysteresis parameters (Table 1) in both the loess-paleosol and red clay unmixing models. In contrast to the loess-paleosol sequence, EM1 in the red clay sequence has lower remanent coercivity (B_cr_), lower coercivity (B_c_) (Table 1) and a more pronounced wasp-waisted shape (Fig. 3a,c).

The correlation between EM abundance (in terms of magnetization) and mass-specific magnetic susceptibility (χ) is presented in Fig. 4a,b. Both the EM1 and EM2 abundances exhibit a linear correlation with χ (inverse and positive correlations for EM1 and EM2, respectively). Frequency-dependent magnetic susceptibility (χ_fd_) (a proxy of superparamagnetic particles close to the stable single domain threshold size) also displays a negative (positive) correlation with EM1 (EM2) (Fig. 4c,d).

Figures 5 and 6 show a good agreement between EMs derived from an earlier IRM unmixing model^16^ (Figs 5e,f and 6f,g) and the present hysteresis unmixing (Fig. 5c,d and 6c,e). The IRM and hysteresis models display similar variations between EM2 and χ (and χ_fd_) since ~8 Ma (Figs 5a–c,f and 6a–c,g). Hematite abundance estimated from diffuse reflectance spectra (DRS)^21^ and EM1 abundance derived from hysteresis unmixing exhibit a consistent trend in the red clay sequence, with a higher content at the bottom of the sequence and a lower content at the top (Fig. 6d,e).

## Discussion and Conclusions

The loess-red clay deposits on the CLP are considered to consist of material originating from the arid Gobi or northwestern deserts in China^22^,^23^,^24^,^25^,^26^. Recent studies, however, emphasize contributions from fluvial dry beds and alluvial fans in front of mountain belts^27^,^28^,^29^,^30^,^31^,^32^,^33^. Importantly, the eolian sediments on the CLP contain a quasi-continuous paleoclimatic record from the Miocene to the Quaternary^4^,^9^,^10^,^19^,^34^,^35^,^36^,^37^,^38^. After dust deposition on the CLP, increased precipitation and elevated temperatures during interglacials promoted the production of a fine-grained pedogenic magnetic component with high χ, while low precipitation and decreased temperatures during glacials favored preservation of the low χ coarse-grained eolian magnetic component^10^,^39^,^40^. Given this model, consideration of two magnetic components (detrital and pedogenic) should explain the observed magnetic properties of the loess-paleosol and red clay sequences. This is confirmed by unmixing of IRM curves^16^, the hysteresis loop unmixing presented here (Fig. 3) and the characteristics of the end-members themselves (Table 1). Previous studies have focused on paleoclimatic information provided by ferrimagnetic minerals^1^,^11^,^41^,^42^,^43^. Such an approach, however, ignores any paleoclimatic information carried by the high coercivity magnetic mineral component. Reconstructions based on high coercivity minerals have an important role to play in testing the validity of CLP climatic histories derived from lower coercivity ferrimagnetic minerals^17^. Using hysteresis unmixing we are able to discuss the magnetic data in the context of both ferrimagnetic and high-coercivity minerals.

The abundance of EM2 in both the loess-paleosol and red clay sequences is positively correlated with χ and χ_fd_ (Figs 4b,d, 5a–c and 6a–c). This suggests that EM2 corresponds mainly to a pedogenic magnetic component. In line with this suggestion, we argue that the strong correlation between EM2 and χ_fd_ indicates that this component reflects contributions from fine-grained (i.e. particles at the superparamagnetic/stable single domain boundary) rather than coarse-grained (i.e. multidomain) material^19^ (Fig. 4d). The EM2 hysteresis loop does not close until ~500 mT and we attribute this to a contribution from high coercivity minerals. However, given the correlation between EM2 and χ_fd_, we feel that any contribution from a high-coercivity component is likely to be small and importantly the covariation with the concentration of pedogenic ferrimagnetic grains is consistent with a recently proposed hematite formation pathway (ferrihydrites to maghemite and then to hematite)^44^,^45^,^46^.

IRM unmixing results^16^ reveal that both the loess-paleosol and red clay samples have a pedogenic component with peak coercivity of ~21 mT. The similarly narrow hysteresis loops and characteristic parameters of EM2 (Fig. 3b,d and Table 1) in the loess-paleosol and red clay are consistent with the IRM unmixing results. These findings reinforce the earlier observation that the Quaternary loess-paleosol and late Miocene-Pliocene red clay may have experienced a similar magnetic enhancement process^7^,^19^,^47^ (i.e., magnetic enhancement is caused by precipitation-induced production of the nano-scale ferrimagnetic grains) and that East Asian monsoon precipitation increased from 4.5 to 2.6 Ma, before decreasing from 2.6 to 1.2 Ma as Northern Hemisphere glaciations intensified^40^.

EM1 corresponds to a “wasp-waisted” hysteresis loop in both the loess-paleosol and red clay sequences (Fig. 3a,c) and its abundance exhibits an inverse relationship with χ and χ_fd_ (Fig. 4a,c), similar to the IRM-based EM1^16^. In the case of IRM unmixing^16^, this end-member was not attributed to a pedogenic origin but to a detrital origin with temperature changes exerting control over the degree of particle oxidation. Although it is feasible to attribute hysteresis-based EM1 to the same mechanism, we note that the content of hematite in the red clay sequence decreased from ~4.5 to 2.6 Ma (Fig. 6d) and a previous study shows that the content of hematite decreased further after 2.6 Ma^21^. Thus, we argue that variation in hematite content can also contribute to the observed EM1 component variations from the bottom to the top of the red clay and from the red clay into the loess-paleosol sequence. Because the production of hematite requires higher temperatures, the decreased contribution of EM1 from the bottom to the top of the red clay and less apparent wasp-waisted shape of EM1 in the Quaternary loess sequence indicate that the CLP experienced a cooling trend since the late Pliocene. This interpretation is consistent with the concept that the shape of wasp-waisted hysteresis loop results from mixtures of magnetic grains that have widely different coercivities. Thus, we argue that EM1 variation reflects both oxidation degree of detrital ferrimagnetic grains and relative content of pedogenic hematite. Coarse detrital hematite input may also contribute to the magnetic mineral assemblage, but it is hard to imagine that the concentration of this component varies sufficiently to control hysteresis loop shape. Fine-grained hematite formed in the source regions by weathering could contribute to the behavior of the hysteresis loops, however, we cannot separate this hematite component from the pedogenic hematite formed *in situ* on the Loess Plateau. Pedogenic magnetite/maghemite particles have a rather broad grain-size distribution, but as shown in previous studies, the grain size distribution appears to be almost independent of the degree of pedogenesis^48^. Thus, it is difficult to attribute the wasp-waisted behavior to mixing of magnetite/maghemite with different grain sizes.

In summary, the hysteresis loop unmixing results are generally consistent with the results of the earlier IRM unmixing. But the wasp-waisted form and abundances of the hysteresis-based end-members reveal that the content of hematite decreased from the bottom to the top of the red clay and from the red clay to the loess-paleosol sequence, indicating climatic cooling since the late Pliocene.

### Study site and methods

**Figure 1:**
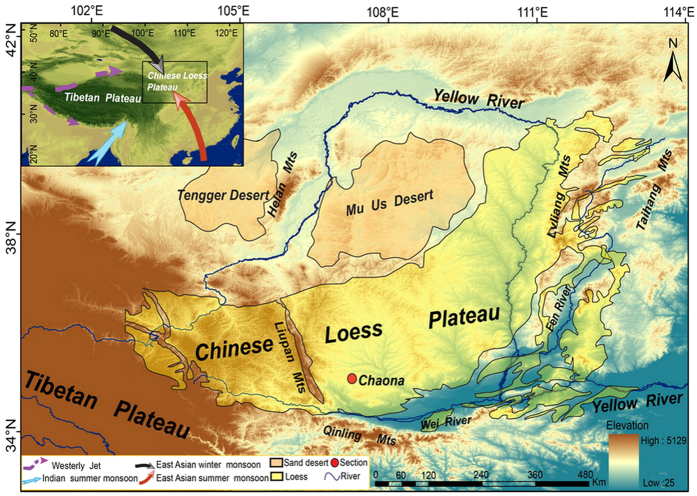
Map showing the physical geography of the Chinese Loess Plateau and pattern of modern Asian atmospheric circulation. The main map corresponds to the area within the rectangle in the index map. ArcGIS 9.3 was used to create the base map and the SRTMDEMUTM 90 M data were obtained from: http://www.gscloud.cn/.

**Figure 2:**
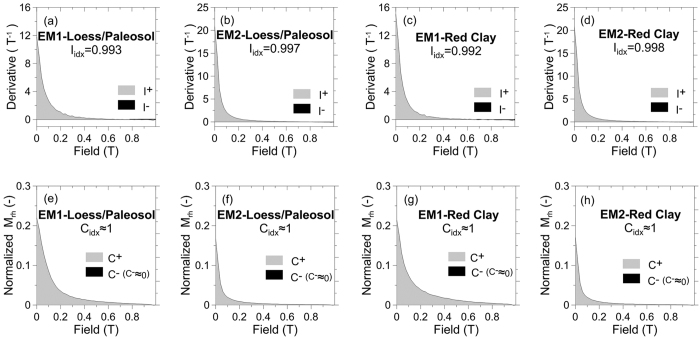
The index of I_idx_ for two end-members in the loess-paleosol (**a**,**b**), and red clay (**c**,**d**) samples. For positive hysteresis fields, the regions of the branch with positive (light shading) and negative (dark shading) derivatives provide the I_idx_ metric, which quantifies the overall monotonicity of the hysteresis branches. (**e**–**h**) (**e**,**f** and **g**,**h** represent the loess-paleosol and red clay samples, respectively) correspond to the C_idx_ index that quantifies the extent of hysteresis branch crossing based on the regions of the curve above (light shading) and below (dark shading) zero. I_idx_ and C_idx_ are defined fully by Heslop and Roberts (2012)^20^.

**Figure 3:**
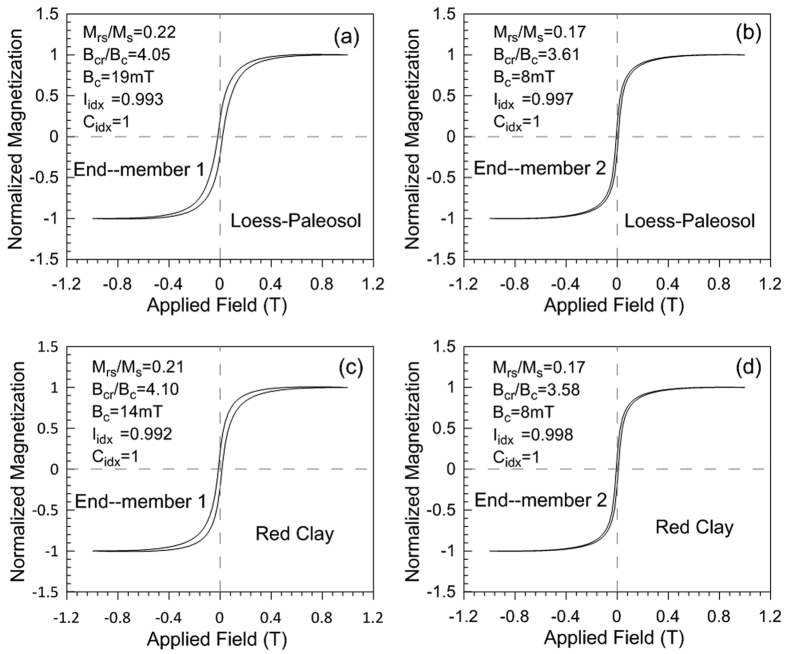
End-members obtained by unmixing hysteresis data from the Chaona section. (**a**–**d**) correspond to loess-paleosol and red clay end-members, respectively. For each end-member loop, the coercivity (B_c_), the ratios of saturation remanent magnetization to saturation magnetization (M_rs_/M_s_) and the median field of the remanent component of the loop to the coercivity (B_cr_/B_c_) are shown (the monotonicity index, I_idx_, and crossover index, C_idx_, are also given).

**Figure 4:**
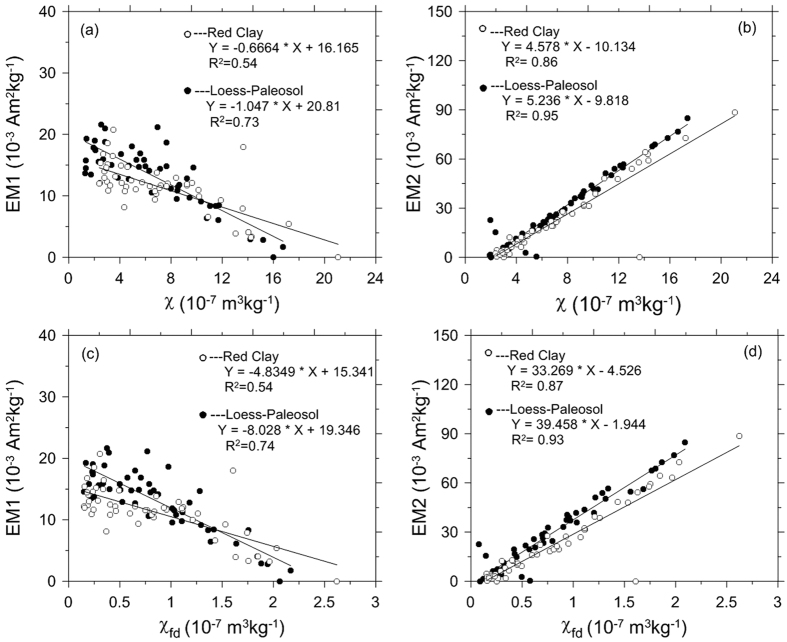
Comparison between end-member (EM1 and EM2) abundances, mass-specific magnetic susceptibility (χ) and frequency-dependant magnetic susceptibility (χ_fd_) for the Chaona section. Filled and open circles correspond to loess-paleosol and red clay samples, respectively.

**Figure 5:**
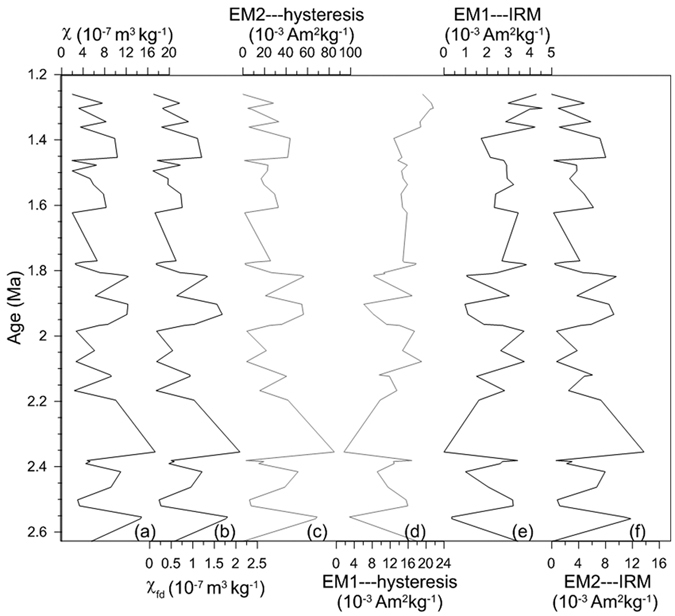
Chaona loess-paleosol sequence (~2.6–1.2 Ma) variations in (**a**) mass-specific magnetic susceptibility (χ), (**b**) frequency-dependent magnetic susceptibility (χ_fd_), (**c**) EM2 abundance (hysteresis-based), (**d**) EM1 abundance (hysteresis-based), (**e**) EM1 abundance (IRM-based) and (f) EM2 abundance (IRM-based). EM1---hysteresis and EM2---hysteresis are the end members derived from the hysteresis unmixing model, while EM1---IRM and EM2---IRM are from the IRM unmixing model.

**Figure 6:**
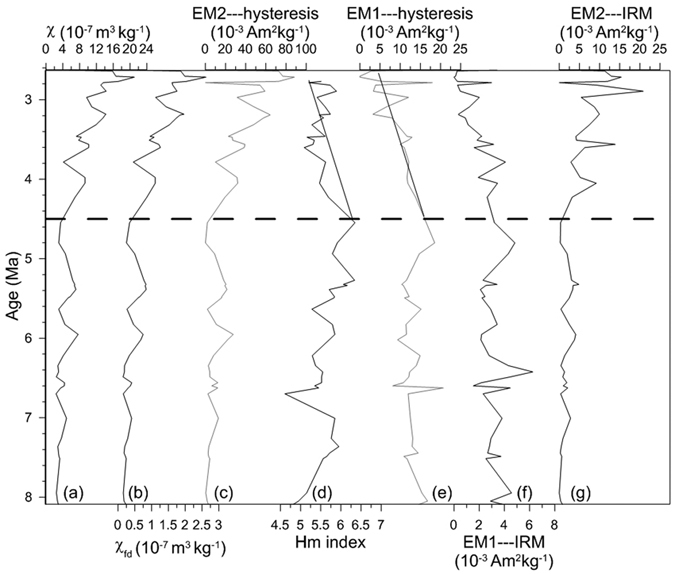
Chaona red clay sequence (~8–2.6 Ma) variations in (**a**) mass-specific magnetic susceptibility (χ), (**b**) frequency-dependent magnetic susceptibility (χ_fd_), (**c**) EM2 abundance (hysteresis-based), (**d**) relative proportion of hematite (DRS-based Hm index), (**e**) EM1 abundance (hysteresis-based), (**f**) EM1 abundance (IRM-based) and (**g**) EM2 abundance (IRM-based). EM1---hysteresis and EM2---hysteresis are the end-members derived from the hysteresis unmixing model, while EM1---IRM and EM2---IRM correspond to the IRM unmixing model. The gray dotted line corresponds to the ~4.5 Ma climate transition on the CLP.

The Chaona section (107°12′ E, 35°6′ N) is located to the east of the Liupan Mountains within Lingtai County, in the central CLP (Fig. 1). The section is ~300 m thick, with the upper 175 m comprising the loess-paleosol sequence and the underlying 125 m comprising the red clay sequence. Paleomagnetic studies indicate that the basal age of the Chaona section is about 8.1 Ma^49^,^50^. For this study, 100 samples were selected (50 samples from loess-paleosol sequence and 50 samples from red clay sequence). The selected loess and red clay samples were ground into powder for hysteresis and diffuse reflectance spectra (DRS) analysis.

Mass-specific magnetic susceptibility (χ) and frequency-dependent magnetic susceptibility (χ_fd_) of the Chaona samples are based on previous work^16^. Diffuse Reflectance Spectra (DRS) were measured with a Purkinje General TU1901 UV-VIS spectrophotometer from 400 nm to 700 nm (1 nm interval), and the area at 555–575 nm in the spectrum first derivative was used to indicate the relative hematite content, termed the “Hm index”^21^. Hysteresis loops were measured on a Princeton Measurements Corporation 3900 VSM (1 T maximum field) and corrected for para/diamagnetic contributions. In addition, the measured loops were adjusted for both vertical and horizontal offsets and corrected for drift^51^.

To analyze the hysteresis loops we use a recently developed unmixing algorithm^20^. As in IRM unmixing^15^, the linear mixing system is represented as *X* = *AS* + *E*, where X contains the average of the rotated lower and upper branches of measured hysteresis loops – yielding *n* rows (1 per sample) and *l* columns (1 per applied field). A, S and E are the abundances (size *n* × *p*), end member signatures (*p* × *l*) and error matrices (*n* × *p*), respectively. In a physically realistic mixing model, the abundances must be non-negative, thus A ≥ 0. To ensure conservative mixing a supplementary constraint is imposed, namely A1_p_ = 1 (where 1_p_ is a column vector of length *p* composed of ones), which specifies that each row of A must sum to unity (this requires the hysteresis loops to be normalized to their maximum magnetization prior to unmixing). The number of end-members (EMs) to include in the mixing model is initially estimated by principal component analysis^20^. A more solid model complexity selection is provided through monotonicity (I_idx_) and crossover (C_idx_) metrics^20^. These indexes are estimated through bootstrap iterations (usually 10^3^) involving the selection of *n* rows from the data matrix, *X*. Physically realistic EMs are indicated by values of I_idx_ and C_idx_ close to 1. For a more detailed description about the mathematical approach of the algorithm we refer the reader to Heslop and Roberts (2012)^20^.

## Additional Information

**How to cite this article**: Zhang, R. *et al*. Unmixing hysteresis loops of the late Miocene–early Pleistocene loess-red clay sequence. *Sci. Rep.* **6**, 29515; doi: 10.1038/srep29515 (2016).

## Figures and Tables

**Table 1:** End-member hysteresis parameters.

End-member	M_rs_	M_s_	M_rs_/M_s_	B_c_(T)	B_cr_(T)	B_cr_/B_c_
EM1-Red Clay	0.21	1	0.21	0.014	0.058	4.10
EM2-Red Clay	0.17	1	0.17	0.008	0.029	3.58
EM1-Loess/Paleosol	0.22	1	0.22	0.019	0.075	4.05
EM2-Loess/Paleosol	0.17	1	0.17	0.008	0.029	3.61
